# Vibration assisted electron tunnelling in COVID-19 infection using quantum state diffusion

**DOI:** 10.1038/s41598-024-62670-3

**Published:** 2024-05-27

**Authors:** Muhammad Waqas Haseeb, Mohamad Toutounji

**Affiliations:** 1 Department of Physics, United Arab Emirates University, Al-Ain, UAE; 2 Department of Chemistry, United Arab Emirates University, Al-Ain, UAE

**Keywords:** Open quantum dynamics, Stochastic equation, Quantum Biology, Biological physics, Theoretical physics, Perturbations

## Abstract

The spread of the COVID-19 virus has become a global health crisis, and finding effective treatments and preventions is a top priority. The field of quantum biology primarily focuses on energy or charge transfer, with a particular emphasis on photosynthesis. However, there is evidence to suggest that cellular receptors such as olfactory or neural receptors may also use vibration-assisted electron tunnelling to enhance their functions. Quantum tunnelling has also been observed in enzyme activity, which is relevant to the invasion of host cells by the SARS-CoV-2 virus. Additionally, COVID-19 appears to disrupt receptors such as olfactory receptors. These findings suggest that quantum effects could provide new insights into the mechanisms of biological systems and disease, including potential treatments for COVID-19. We have applied the open quantum system approach using Quantum State Diffusion to solve the non-linear stochastic Schrödinger equation (SSE) for COVID-19 virus infection. Our model includes the mechanism when the spike protein of the virus binds with an ACE2 receptor is considered as dimer. These two entities form a system and then coupled with the cell membrane, which is modelled as a set of harmonic oscillators (bath). By simulating the SSE, we find that there is vibration-assisted electron tunnelling happening in certain biological parameters and coupling regimes. Furthermore, our model contributes to the ongoing research to understand the fundamental nature of virus dynamics. It proposes that vibration-assisted electron tunneling could be a molecular phenomenon that augments the lock-and-key process for olfaction. This insight may enhance our understanding of the underlying mechanisms governing virus-receptor interactions and could potentially lead to the development of novel therapeutic strategies.

## Introduction

The open systems paradigm acknowledges that no system operates in isolation and utilizes statistical methods and approximations to accommodate for unknown and uncontrollable variables. This approach has been immensely successful in applying theoretical concepts to practical predictions and has resulted in numerous technological advancements. Quantum biology has been around since the early days of quantum mechanics^1^, and it focuses on the interaction between light and living matter, as opposed to the interaction of light and nonliving matter. The study of photosynthesis, a crucial process in biology, is an example of this. The properties of viruses, which straddle the line between living and nonliving systems, make them a unique case study for quantum biology. Some research has already been done on the intersection of quantum mechanics and viruses, including the engineering of a virus to enhance energy transport and the use of quantum dots to label viral proteins for better imaging^2^. Quantum dots have even been suggested to have antiviral properties^3–5^. Furthermore, endeavors have been undertaken to replicate the life cycle of a virus through the utilization of quantum simulation techniques^6^.

The domain of quantum biology is committed to the systematic examination of the plausible influence arising from intricate quantum phenomena, including coherence, entanglement, and tunneling, within the framework of biological systems^7^. The field has gained increasing attention, and energy and charge transfer have emerged as a central focus of research.The phenomenon of coherent energy and charge transfer has been observed to wield significance within photosynthetic networks, representing a prominent exemplar of quantum effects application within biological systems^8^. Additionally, tunnelling in enzymes has been observed for several decades and is also significant in the context of charge transfer. The consideration of quantum tunneling has been put forth as a conceivable mechanism that could underlie the activation of olfactory receptors by olfactants. These olfactory receptors belong to the G-protein coupled receptor (GPCR) family and bear similarity to rhodopsin-like receptors. Their involvement extends across various critical physiological processes, including the regulation of inflammatory responses and the facilitation of neurotransmitter binding^9^. The vibration-assisted electron tunnelling has been identified as a promising method for investigating the binding of neurotransmitters. In some cases, electron tunnelling has been considered an alternative mechanism to the lock-and-key model, which is based on the shape of the receptor binding^10^.

**Figure 1:**
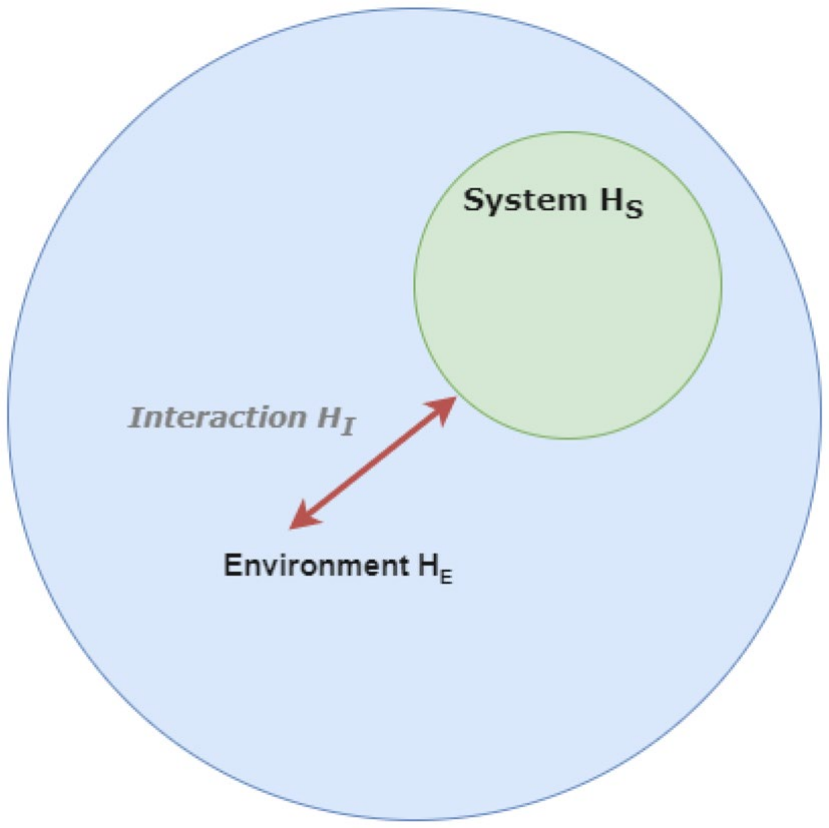
This figure presents a graphical representation of an open quantum system. The central quantum system is depicted as a blue circle, while its encompassing environment is portrayed as a green cloud. The quantum system’s intrinsic dynamics are determined by its Hamiltonian, and the encompassing environment contains multiple degrees of freedom influencing the system. Arrows demonstrate the flux of energy and data between the quantum system and its surroundings, emphasizing the interconnectedness inherent to open quantum systems. It is pivotal to factor in the interplay between the quantum system and its environment for a holistic understanding, given that open quantum systems often display intricate behaviors not observed in isolated systems.

Quantum biology is a field that investigates the impact of quantum effects on biological systems, including the phenomenon of quantum tunnelling, in which particles pass through barriers that classical mechanics would consider impenetrable. Proton and electron tunnelling have been implicated in DNA mutations and enzymatic reactions^11^, G-protein coupled receptors (GPCRs) represent a class of integral membrane proteins that play a pivotal role in various physiological processes, including olfaction and neurotransmitter binding. Recent research has suggested that the functioning of GPCRs may involve quantum mechanical phenomena, adding an intriguing dimension to the burgeoning field of quantum biology. Of particular interest is the connection between GPCRs and rhodopsin, a photoreceptor protein found in the retina^12^. Rhodopsin, a prototypical GPCR, contains the light-sensitive chromophore retinal, bound within an opsin protein. The interaction between retinal and opsin leads to the activation of the visual signaling cascade in response to photon absorption, initiating the process of vision. The possibility that quantum effects underlie aspects of GPCR function, especially in the context of light detection by rhodopsin, underscores the intricate interplay between classical biochemical processes and quantum phenomena in biological systems, offering new avenues for exploration in the realm of quantum biology.Futhermore, chromophores, which are integral to photosynthesis, play a role in redox activity in biological materials, and the vibrational or spin states of proteins may be linked to electronic states in a beneficial way, potentially increasing energy or charge transfer^13,14^. Considering the use of membrane-embedded proteins by SARS-CoV-2 to invade host cells, investigating the role of quantum effects in charge transfer could be useful.

In exploring the dynamics of a quantum system, it’s vital to account for its interaction with the surrounding environment or reservoir. Such incorporation becomes feasible through the open quantum system (OQS) framework. This framework integrates the Hamiltonian of the primary system, the external environment, and their mutual interaction, as shown in Fig. 1. A popular method for delving into these dynamics involves the master equation technique. The key focal point is the central system’s reduced density matrix, derived by tracing over the reservoir’s degrees of freedom. Equations like the Nakajima-Zwanzig equation^15,16^, Redfield’s master equation^17^, the Lindblad formula^18^, Hu-Paz-Zhang equation^19^, and the broad non-Markovian time-local master equation^20^ dictate the evolution of this matrix. By employing the master equation, it’s possible to compute time evolving expectation values for any operator in the *S* system’s Hilbert space, solving a set of regular differential equations.

The conventional approach to studying vibrational-assisted electron transfer, grounded in the semi-classical theory using Fermi’s Golden Rule, offers a foundational method for calculating transition rates only in systems with weak electronic coupling. However, research performed in^21^ challenges the sufficiency of this semi-classical framework in accurately predicting the dynamics of olfactory receptors, particularly in low-frequency environments where semi- classical theories were presumed most effective. Their work employing a polaron master-equation approach illuminates how incorporating strong dissipation into the model not only reconciles the discrepancies with classical rate predictions but also enhances the system’s switching capabilities beyond the dissipationless scenario. Critically, this approach reveals limitations in the semi-classical theory’s ability to resolve odorant frequencies under strong dissipation conditions, suggesting that transcending these classical bounds can significantly improve selectivity for specific odorant modes, despite potential increases in background noise.

An alternative methodology employed for exploring the dynamics of open quantum systems (OQS) is the stochastic Schrödinger equation (SSE), as discussed by Bouten et al. (2004)^22^. The motivation to use SSE is that the system’s reduced density matrix, achieved by tracing out environmental degrees of freedom, is depicted through an ensemble of continuous trajectories without any approximation. These trajectories represent various outcomes of the driving complex Gaussian process. This approach allows for an expansion based on the ratio of the environmental correlation time to the system’s typical time scale. The leading order of this expansion, the Markovian approximation is obtained, while subsequent orders provide the non-linear non-Markovian stochastic Schrödinger equation (SSE). Specifically, our analysis prioritizes the initial correction, aligning with the zeroth-order Markovian SSE, which is fundamental for capturing the system’s evolution accurately. Notably, the non-Markovain version of SSE is particularly effective, offering a true representation of the system’s evolution. SSE easily can be extended to Markovain to non-Markovain regimes and works well in the strong coupling strength as well. This method shines when dealing with bosonic environments, thanks to its unmatched applicability, ease of implementation, and efficiency in numerical simulations as compared to Master equation formulations^23^. Notably, for Markovian systems, the outcomes deduced from the SSE are found to align with those arising from the corresponding master equation in the Lindblad formalism.

This paper is systematically divided into five main sections. Section “Introduction and Background,” sets the scene by discussing the overall context of the research, its significance, and a brief review of prior related works. In Section “Methodology” we delve into the specifics of our research process, touching upon the methods of data collection, analytical tools and techniques employed, and any inherent limitations of our approach. Section “Numerical Findings,” is dedicated to the presentation, analysis, and interpretation of our primary results, juxtaposed with findings from preceding studies for comparative insights. We wrap up our discourse in Section “Conclusion” summarizing the key takeaways, their broader implications, and offering pointers for prospective research endeavors. Finally, Section “Appendix,” in which complete derivation of the non-linear non-Markovian stochastic Schrödinger equation (SSE) is presented.

1 $$\begin{aligned} H_{\text {T}}=H_S+\gamma \left( L \sum _{k} g_k b^+_k+L^+\sum _{k} g_k^* b_k\right) +H_R \end{aligned}$$

The Hamiltonian $H_S(R)$ describes the system-reservoir energy. $b_k$ and $b^k$ are annihilation and creation operators in the bosonic environment. $\gamma$ is a variable perturbation parameter. $g_k(g_k^*)$ and $\omega _k$ represent coupling strength and eigen frequency of the *k*-th mode of the environment. These parameters influence the system-reservoir interaction and dynamics.

## Background

### Linear stochastic Schrodinger equation

The dynamics of a quantum open system, characterized by its Hamiltonian, is dictated by the Schrödinger equation.

2 $$\begin{aligned} \frac{d}{d t}| \Psi (t)\rangle = -\frac{i}{\hbar } H_{\text {T}} | \Psi (t) \rangle \end{aligned}$$

The system is coupled to a reservoir comprised of set of bosonic oscillators, as represented by the Hamiltonian $H_R= \sum _{k} \omega _k b_k^+b_k$. The reservoir is best described using the Bargmann coherent state basis, denoted as $|z\rangle = \bigotimes _k |z\rangle$. This basis is a tensor product, bringing together all the distinct oscillators in the environmental configuration. Within this context, the notation $|z\rangle$ is specifically described by the relationship $b_k |z\rangle = z_k |z\rangle$, pointing to the kth mode in the environment. Given this basis, one can view the system’s state vector as a stochastic path or trajectory. Its dynamics in the interaction picture, follows a formula where the standard quantum unit $\hbar$ is taken as unity.

3 $$\begin{aligned} \frac{d}{d t} \langle z | \Psi (t) \rangle = -i \langle z | H_{\text {T}} | \Psi (t) \rangle =(-i H_S + \gamma L^\dagger z^* t) \langle z | \Psi (t) \rangle - i \gamma \sum _k g_k^* e^{-i \omega _k t} \langle z | b_k | \Psi (t) \rangle \end{aligned}$$

Here, the complex Gaussian noise is represented as $z_t^* = -i\sum _k g_k z_k^* e^{i \omega _k t}$, and it conforms to the stipulated auto-correlation function $\alpha (t,s) = \mathscr {M}[z^*(t)z(s)]$. The operator denoted by $\mathscr {M}$ encompasses an ensemble average over the classical noise emanating from $z_t$, and is symbolized as $\mathscr {M} = \int d\mu (z)[\cdot ]$. The integration measure $d\mu (z) = \frac{d^2z}{\pi } e^{-|z|^2}$ encapsulates the classical probabilistic distribution of *z*, while $\int d\mu (z) |z\rangle \langle z| = I$ asserts the completeness condition. Moreover, the time-evolution operator *U*(*t*), governing the interplay between the system and the environment, is defined through $|\Psi (t)\rangle = U(t)|\Psi (0)\rangle$. This operator adheres to an evolution equation that characterizes the dynamical progression.

4 $$\begin{aligned} \frac{d}{d t}U(t) = -i\left( H_S + \gamma L \sum _k g_k b_k^\dagger e^{i\omega _k t} + \gamma L^\dagger \sum _k g_k^* b_k e^{-i\omega _k t}\right) U(t) \end{aligned}$$

and following the procedure mentioned in (Appendix) the Stochastic Schrodinger Equation is

5 $$\begin{aligned} \frac{d}{d t} \psi _t(z) = \left( -iH_S + \gamma L z_t - \gamma ^2 L^\dagger \int \limits _0^t ds \alpha (t,s) \hat{X}(t, s, z)\right) \psi _t(z) \end{aligned}$$

by defining an operator $\hat{X}(t,s,z)$

6 $$\begin{aligned} \hat{X}(t, s, z)\psi _t \equiv \dfrac{\delta \psi _t}{\delta z_s} \end{aligned}$$

Here $X(s, s, z) = L$. The consistency condition is used to calculate the time-dependence of *X*(*s*, *s*, *z*).

7 $$\begin{aligned} \frac{d}{dt} \frac{\delta }{\delta z} \psi _t = \frac{\delta }{\delta z} \frac{d}{dt} \psi _t \end{aligned}$$

The equation labeled as (Eq. 5) is governed by linearity. In the case of an infinite heat bath, the norm $||\psi _t||$ of the solution converges to zero with a probability of one, while it diverges to infinity with a probability of zero. This non-Markovian development aligns with the principles of the reduced density operator technique. By aggregating the outcomes from (Eq. 5) relative to the noise $z_t$ , we can accurately reconstruct the corresponding density matrix of the system.

8 $$\begin{aligned} \rho _t \equiv \textrm{Tr}_ {\textrm{env}} \left[ e^{-iH{\textrm{T}}t} |\psi _0\rangle \langle \psi _0| \otimes \rho _{\textrm{env},0} e^{iH_{\textrm{T}}t} \right] = \mathscr {M} \left[ |\psi _t(z)\rangle \langle \psi _t(z)|\right] . \end{aligned}$$

### Non-linear non-markovain quantum state diffusion

The non-Markovain Unraveling of Quantum State Diffusion (QSD) predicated on states with normalization

9 $$\begin{aligned} \tilde{\psi }_t(z) = \frac{\psi _t(z)}{||\psi _t(z)||} \end{aligned}$$

could be achieved using the Girsanov transformation^24^.

10 $$\begin{aligned} \frac{d}{dt}\tilde{\psi }_t = -iH_S\tilde{\psi }_t + (L - \langle L\rangle _t)\tilde{\psi }_t \tilde{z}_{t} - \int \limits _0^t ds \alpha (t,s) \langle (L^\dagger - \langle L^\dagger \rangle _{s}) \hat{X}(t,s,{\tilde{z}_t}) - (L^\dagger - \langle L^\dagger \rangle _{s})\hat{X}(t,s,{\tilde{z}_t}) \rangle \tilde{\psi }_t \end{aligned}$$

Where ${\tilde{z}_t}$ is the shifted noise,

11 $$\begin{aligned} {\tilde{z}_t}=z_t+\int \limits _0^t ds \alpha (t,s) \langle L^\dagger \rangle _{s} \end{aligned}$$

and $\langle L\rangle _{s}=\langle \tilde{\psi }_t| L |\tilde{\psi }_t \rangle$ is the quantum average.

The non-linear non-markovain QSD equation can have the compact form by introducing $\Delta _t(A)=A-\langle A\rangle _t$

12 $$\begin{aligned} \frac{d}{dt}\tilde{\psi }_t = -iH_S\tilde{\psi }_t + \Delta _t(L)\tilde{\psi }_{t}z^{*}_t - \Delta _t(L^{\dagger })\bar{X}(t, \tilde{z})\tilde{\psi }_t + \Delta _t(L^{\dagger })\bar{X}(t, \tilde{z})_t\tilde{\psi }_t \end{aligned}$$

Where

13 $$\begin{aligned} \bar{X}(t, z) = \int \limits _{0}^{t} \alpha (t,s)\hat{X}(t,s,z)ds \end{aligned}$$

The (Eq. 10 or  12) is fundamental non-Markovain Quantum state diffusion and in the next section perturbative treatment start with this equation. Numerical simulations of the some of the non-Markovain open quantum system dynamics can be found in this paper.^25^ In this paper we focus on the Markovain dynamics which can be obtained from the (Eq. 10 or  12) by simply replacing $\alpha (t,s)\rightarrow \delta (t-s)$^26^. To obtain the non-Markovian equations, specific assumptions were made. First, the environment exhibits bosonic characteristics, and the initial state of both the system and the environment is distinguishable: $\rho _0 = \rho _S^{(0)} \otimes \rho _{env}^{(0)}$, which remains unaffected by the noise $z_t$. Second, the non-Markovian equations are exclusively derived from the underlying microscopic model, as evident in the Hamiltonian presented in (Eq. 1). By Applying the formal Perturbation theory on operator $\hat{X}(t, s, z)$ using a series expansion in powers of $(t-s)$ (see "Appendix").

14 $$\begin{aligned} \frac{d}{dt}\tilde{\psi }_t= & {} -iH_S\tilde{\psi }_t + \Delta _t(L)\tilde{z}_t - g_0(t)((\Delta _t(L^{\dagger })L - \langle \Delta _t(L^{\dagger })L \rangle _t))\tilde{\psi }_t + ig_1(t)(\Delta _t(L^{\dagger })[H,L] - \langle \Delta _t(L^{\dagger })[H,L]\rangle _t))\tilde{\psi }_t\nonumber \\{} & {} + g_2(t)((\Delta _t(L^{\dagger })[L^{\dagger },L]L - \langle \Delta _t(L^{\dagger })[L^{\dagger },L]\rangle _t))\tilde{\psi }_t \end{aligned}$$

In the realm of non-Markovian Quantum State Diffusion (QSD) equations, the zeroth-order term, obtained through expansion, introduces a crucial magnitude that is contingent upon the typical relaxation rate of the quantum system, symbolized as $\Gamma$. When one delves into the first-order terms, they emerge as corrections characterized by scaling factors of either $\omega \tau$ or $\Gamma \tau$, where $\omega$ signifies the standard system frequency represented by the Hamiltonian *H*, and the composite operator $L^{\dagger }L$ embodies a common system relaxation rate, $\Gamma$. In situations marked by non-Markovian dynamics, wherein the environmental correlation time remains finite but does not exceed the characteristic time scales of the quantum system, it is anticipated that the expanded QSD equation maintains its validity. However, in the limit where the correlation time $\tau$ tends towards zero, the Markovian regime emerges. In this limiting scenario, only the zeroth-order term persists, culminating in the well-established Markov Quantum State Diffusion equation that governs the quantum dynamics for $t > 0$. This delineation underscores the transition from non-Markovian to Markovian behavior in quantum systems and offers valuable insights into the conditions under which each regime prevails.

The Quantum State Diffusion (QSD) equation, as represented in (Eq. 14), is a fundamental tool in quantum dynamics. It is explicitly formulated by considering the Hamiltonian governing the system, the Lindblad operator characterizing the system’s interaction with its environment, and the commutators associated with these components. Once the physical model is rigorously defined, these mathematical elements can be derived systematically. However, a crucial aspect in this framework lies in determining the coefficients $g_i(t)$, which play a central role in describing the dynamics of the quantum system. These coefficients are intrinsically linked to the environmental correlation function $\alpha (t,s)$, which encapsulates the quantum noise stemming from the environment. Notably, in cases where the system is subjected to Ornstein-Uhlenbeck noise, characterized by its exponential correlation function, the behavior of the coefficients $g_i(t)$ assumes a distinctive form. These insights into the determination of $g_i(t)$ are of paramount importance, as they are indispensable for the practical application of the QSD equation in quantum dynamics, facilitating a deeper understanding and control of quantum systems subjected to environmental influences.

15 $$\begin{aligned} \alpha (t, s) = \frac{\eta }{2} e^{-\eta |t-s|} \end{aligned}$$

The finite environmental memory or correlation time is defined as $\eta ^{-1} = \tau$, where $\tau$ is the time scale on which the coefficients $g_i(t)$ change. The equation indicates that opting for this specific $\eta$ leads to a Lorentzian spectral profile. As $\eta$ approaches infinity, the Ornstein-Uhlenbeck noise converges to basic complex white noise.

16 $$\begin{aligned} \alpha (t, s) = \delta (t-s) \end{aligned}$$

For the Ornstein-Uhlenbeck process, the coefficients $g_i(t)$ take the following form:

17 $$\begin{aligned} g_0(t)= & {} \int \limits _{0}^{t} ds_1 \int \limits _{0}^{s_1} ds_2 \alpha (s_1,s_2) = \frac{1}{2\eta }(1-e^{-\eta t}) \end{aligned}$$

18 $$\begin{aligned} g_1(t)= & {} \frac{1}{2\eta } \left( 1 - e^{-\eta t} - \eta t e^{-\eta t} \right) \end{aligned}$$

19 $$\begin{aligned} g_2(t)= & {} \frac{1}{4\eta } \left( 1 - e^{-\eta t} - \eta t e^{-\eta t} - \frac{1}{2\eta ^2} (1-e^{-\eta t})^2 \right) \end{aligned}$$

In the long-time limit where *t* is much larger than the environmental correlation time $\tau$, the coefficients $g_0$, $g_1$, and $g_2$ of the non-Markovian QSD (Eq. 14) become constant. Specifically, $g_0$ is equal to 1/2, while $g_1$ and $g_2$ are equal to $1/2\eta$ and $1/4\eta$, respectively, where $\eta$ is the inverse of the environmental correlation time $\tau$. Furthermore, $g_0$ is of the order one, while $g_1$ and $g_2$ are of the order of $\tau$. In the Markov limit where the environmental correlation time $\tau =\eta ^{-1}$ goes to zero, the coefficients $g_1(t)$ and $g_2(t)$ become zero for $t>0$, while $g_0(t)$ approaches a constant value of 1/2. This means that our non-Markovian QSD (Eq. 14) reduces to the standard Markov equation, which is a simpler equation commonly used in quantum mechanics to describe the evolution of open quantum systems under the influence of a Markovian environment^23^.

To write the equation in Stratonovich form, we need to express the stochastic integral $\Delta _t(L) \tilde{z}_t$ in terms of a Stratonovich integral. This can be done using the following relation :

20 $$\begin{aligned} \Delta _t(L) \circ \tilde{z}_t = \frac{1}{2}(\Delta _t(L)\circ \tilde{z}_t + \tilde{z}_t \circ \Delta _t(L)) \end{aligned}$$

Substituting this in the given equation, we obtain

21 $$\begin{aligned} \frac{d}{dt}\tilde{\psi }_t = -iH\tilde{\psi }_t + \Delta _t(L)\tilde{\psi }_t \circ (z_t + hL^{\dagger }_t) - \frac{1}{2}\Delta _t(L^{\dagger }_t L_t)\tilde{\psi }_t \end{aligned}$$

with $z_t$ the standard complex white noise.

**Figure 2:**
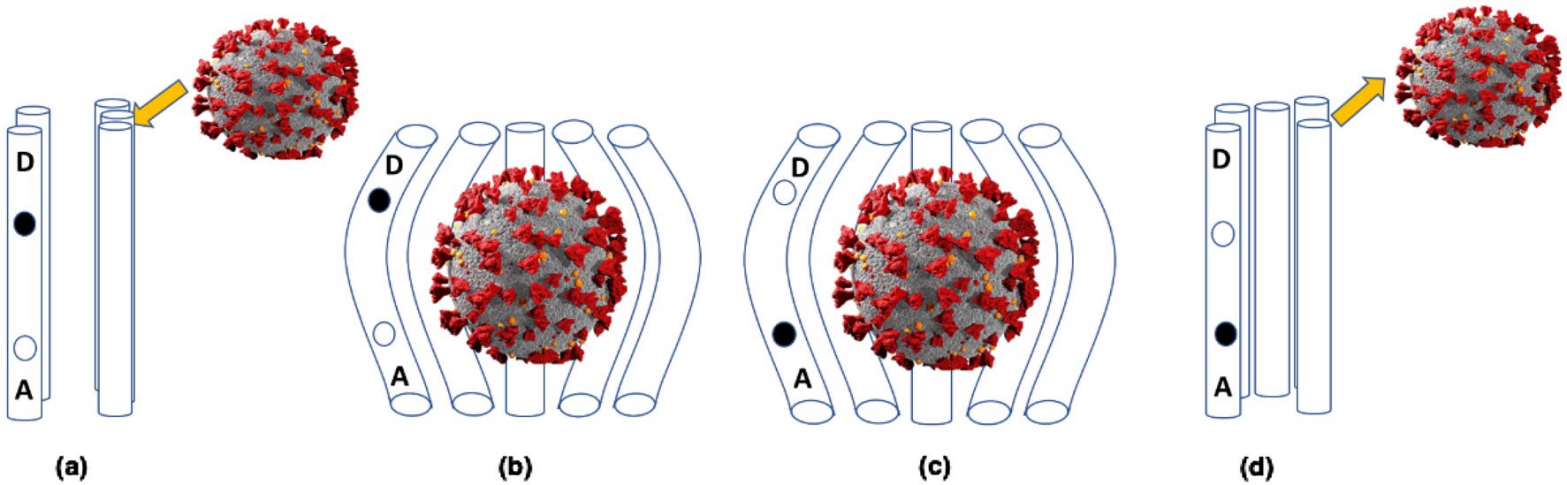
A schematic depiction of intra-protein electron transfer during the interaction of the COVID-19 virus with a cellular receptor. For simplicity, only five trans-membrane helices of the receptor are illustrated as cylinders. (**a**) The virus approaches the receptor, with an electron localized at the donor site *D*. (**b**) The spike protein of the virus attaches to the ligand-binding domain, triggering a conformational alteration in the receptor-virus complex. (**c**) An electron transitions from *D* to *A*, instigating pronounced vibrations in the spike protein. (**d**) Subsequently, the spike protein detaches from the ligand-binding domain. This diagrammatic representation aids in understanding the electron transfer dynamics during the virus-receptor interaction.

## Method

Biological systems are modeled using the open quantum system approach, which takes into account the interaction of the system with its environment^27^. The vibrationally-assisted electron transfer process is modeled utilizing the conventional spin boson model^21,28^. In this framework, a donor-acceptor pair interacts with an environmental bath, which is exemplified by a set of harmonic oscillators^14^. In our methodology, the ligand protein, receptor, and the encompassing environment are treated collectively as a closed system. Drawing inspiration from the model formulated for Olfaction^29^, the open quantum systems approach is employed to probe the linkage between the maximum transfer probability in the ACE2 receptor and its coupling to a vibronic mode inherent to the SARS-CoV-2 spike protein. For a streamlined analysis, the receptor’s representation is condensed to a dimer, with the guiding Hamiltonian explicating the interactions and dynamics of both the system and its environment. A simplified illustration of intra protein electron transfer process is Shown in Fig. 2.

The Hamiltonian for the receptor is

22 $$\begin{aligned} H_{R} = \frac{1}{2} \varepsilon \sigma _z + \frac{1}{2} \Delta \sigma _x \end{aligned}$$

For a dimer isolated from external interaction, the maximum probability of a transition from donor to acceptor is given by^30^

23 $$\begin{aligned} \text {Max}[P_{D \rightarrow A}(t)] = \frac{\Delta ^2}{\Delta ^2 + \epsilon ^2} \end{aligned}$$

where $\varepsilon =\varepsilon _D - \varepsilon _A$ When the energy of the donor and the acceptor are equal, i.e., $\varepsilon _D = \varepsilon _A$, the maximum transfer probability from donor to acceptor $[P_{D\rightarrow A}(t)]$ at time $t_0= \pi /2 \Delta$ is equal to 1. The Hamiltonian of the ligand, in this case the spike protein, is represented as a harmonic oscillator with frequencies $\omega$ associated with the protein:

24 $$\begin{aligned} H_{P}= & {} \omega (b^{\dagger }b+1/2) \end{aligned}$$

25 $$\begin{aligned} H_{R-P}= & {} \sigma _z\sum _{i} \omega _i \gamma _i(b + b^{\dagger }) \end{aligned}$$

The summation is extended to encompass the interaction of the protein with both the donor and the acceptor; however, in the numerical solution, the latter interaction is presumed to be negligible. The degree of coupling between the ligand protein and the receptor is denoted as $\gamma$, with the creation and annihilation operators for the spike protein vibrations represented by *b* and $b^{\dagger }$, respectively, and associated with the frequency $\omega$. The Hamiltonians $H_R$, $H_P$, and $H_{R-P}$ encapsulate the representation of the system within our model concerning the SARS-CoV-2 receptor tunneling phenomenon. Similarly, the membrane environment is characterized by $H_E$, and its interplay with the receptor is approximated as $H_{R-E}$.

26 $$\begin{aligned} H_E= & {} \sum _{E}\omega _E\left( b_E^\dagger b_E + \frac{1}{2}\right) \end{aligned}$$

27 $$\begin{aligned} H_{R-E}= & {} \sigma _z\sum _{i,E} {\omega _E} \gamma _{iE} (a_E + a_E^\dagger ) \end{aligned}$$

In the above equation, the symbol $\gamma _{iE}$ represents the coupling strength between the receptor and its membrane environment, which is weaker than the coupling between the spike protein and the receptor. The Hamiltonians $H_{R-P}$ and $H_{R-E}$ represent competing interactions, where $H_{R-P}$ is crucial for the receptor to recognize the spike protein, and $H_{R-E}$ represents undifferentiated coupling to various environmental vibronic modes near the receptor. The discrimination between the spike protein vibrations and the environmental vibrations is based on their distinct frequencies and the stronger coupling constants. The summation in the equation involves the donor, the acceptor, and all the potential environmental vibrations.

In our problem, the system is composed of the spike protein of the virus and the ACE2 receptor. The Hamiltonian of the system consists of $H_R$, $H_P$, $H_R-P$, and the Lindblad operator is given as $L=\gamma _{iE} \sigma _z$. Therefore, our (Eq. 14) in the Markovian case will have the following form:

28 $$\begin{aligned} \frac{d}{dt}\tilde{\psi }t= & {} -i\left( \frac{1}{2} \varepsilon \sigma _z + \frac{1}{2} \Delta \sigma _x + \omega (b^{\dagger }b+1/2) + \sigma _z\sum {i} \omega _i \gamma _i(b + b^{\dagger })\right) \tilde{\psi }t + \frac{\gamma _{iE}}{2}(\sigma _z- \langle \sigma _z \rangle _t-\langle \sigma _z\rangle )_t)\tilde{\psi }_t \tilde{Z}t \nonumber \\{} & {} + \gamma _{iE}^2 g_0(t) ( \langle \sigma _z \rangle _t \sigma _z - \langle \sigma _z \rangle _t^2) \tilde{\psi }_t \end{aligned}$$

where

29 $$\begin{aligned} {\tilde{Z}t}=Z_t+\int \limits _0^t ds \delta (t-s) \langle \sigma _z^\dagger \rangle {s} \end{aligned}$$

By simulating (Eq. 28) and obtaining the density matrix by ensemble average from (Eq. 8), we find a maximum probability associated with the vibronic mode in the dimer system. The difference in probabilities with and without the vibronic mode takes the following form^31^:

30 $$\begin{aligned} \Delta P= Max[P_{D\rightarrow A}(t)]_{Vibronicmode} - {Max}[P_{D \rightarrow A}(t)]_{without vibronic mode} \end{aligned}$$

## Numerical results

We have derived an expression for the maximum probability of the transition from the donor to the acceptor level as a function of dimer detuning and dimer coupling . By simulating the non-linear Stochastic Schrödinger (Eq. 28) with the Ornstein-Uhlenbeck process as noise and obatining the density matrix by the ensemble average, we have obtained expression for the max probability of electron transition from the donor to the acceptor level. To visualize these results, we have plotted the difference between the maximum probability $$\Delta P= Max[P_{D\rightarrow A}(t)]_{Vibronicmode} - {Max}[P_{D \rightarrow A}(t)]_{without vibronic mode}$$

**Figure 3:**
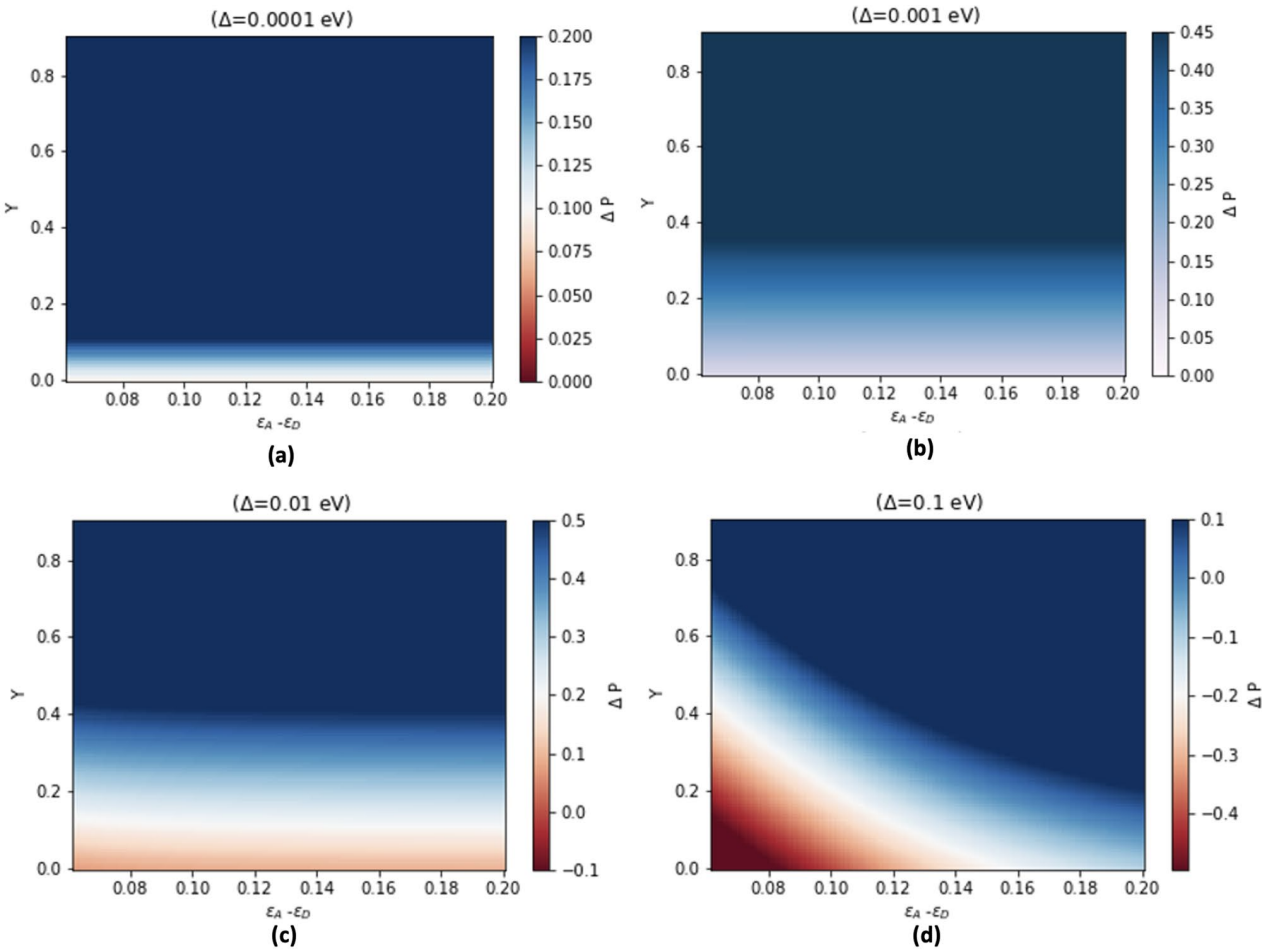
The effect of vibronic mode on the electron transfer probability as a function of dimer coupling strength. (**a**) $\Delta$ = 0.0001, weak coupling limit, showing negligible impact of the vibronic mode. (**b**) $\Delta$= 0.001, slightly stronger coupling, with a small increase in transfer probability. (**c**) $\Delta$ = 0.01, intermediate coupling strength, exhibiting a more pronounced effect of the vibronic mode on the transfer probability. (**d**) $\Delta$ = 0.1, strong coupling limit, revealing a significant negative effect of the vibronic mode on the transfer probability, as indicated by the redder region. The results illustrate the role of the vibronic mode in facilitating electron tunneling across various coupling strengths between the spike protein and the receptor.

We have collected various parameters for our analysis from relevant biological processes through multiple references, in particular Solov’yov et al.’s model for vibration-assisted tunnelling in olfactory receptors^29,32^. These parameters are arranged in Table 1. In the context of olfaction, the energy difference $\varepsilon _{A}$ - $\varepsilon _{D}$ between the redox energies at the D and A sites is significant. Without the presence of an odorant, this energy difference is too large to facilitate thermally assisted electron transfer within a relevant time scale. However, when a suitable odorant binds to the receptor, the vibrational frequency of the odorant, matching $\varepsilon _{A}$ - $\varepsilon _{D}$, enables electron transfer. This occurs as the energy $\varepsilon _{A}$ - $\varepsilon _{D}$, can be released to the vibrational mode of the odorant, which typically exhibits particularly high frequencies. Our model is inspired by the process of olfaction, and we follow the similar line of reasoning in our infection case. This is also reflected in the parameters selection in Table 1. The determination of the tunneling term $\Delta$ between donor (D) and acceptor (A) molecules is inspired by similar physical insights from olfaction processes, with the tunneling parameters presented in Table 1 reflecting the range of parameters that are physically significant in the context of olfaction^21^ . This approach ensures that our model’s parameters are pertinent to biological settings, thereby enhancing the biological accuracy and relevance of our theoretical framework. The coupling between the spike protein and receptor is plotted from weak to strong coupling limits, and the dimer couplings are considered for four values: $\Delta$ = 0.0001, 0.001, 0.01, and 0.1 eV. The frequency of the vibronic mode is obtained from the study that investigated the SARS-COV-2 using Raman spectroscopy^33^. We start our analysis by examining the weak coupling limit ($\Delta$ = 0.0001) and the effect of the vibronic mode on the transfer probability. In Fig. 3, we observe that the vibronic mode has a negligible effect on the transfer probability. This result can be attributed to the fact that the coupling between the spike protein and the receptor is strong as compared to the membrane but coupling between the donor and acceptor is not significant to induce electron tunnelling.

**Table 1:** The parameters for the numerical Simulations^29,33^.

	$\varepsilon _{A}$ - $\varepsilon _{D}$	$\Delta$	$\gamma$	$\omega _{1}$	$\omega _{2}$	$\omega _{3}$
Parameter ranges	500–1700 cm^-1^	0.0001–0.1 eV	0–0.419 eV	1669 cm^-1^	1240 cm^-1^	1000 cm^-1^

As we progress to the next coupling strength value ($\Delta = 0.001$), illustrated in Fig. 3b, the effect of the vibronic mode becomes slightly more pronounced. The transfer probability starts to increase, indicating that the electron tunnelling is affected by the presence of the vibronic mode. However, the overall impact remains limited, as the coupling strength is still relatively weak. At the intermediate coupling strength ($\Delta = 0.01$), shown in Fig. 3c, we observe a more significant effect of the vibronic mode on the transfer probability. The increase in electron tunnelling becomes more noticeable, and the transfer probability reaches a higher value. This result suggests that the vibration-assisted electron tunnelling is becoming more prominent in this regime, and the interaction between the donor and the acceptor is strong enough to facilitate electron transfer. Finally, in the strong coupling limit ($\Delta = 0.1$), depicted in Fig. 3d, the vibronic mode exhibits a significant negative effect on the transfer probability, as indicated by the redder region. This result highlights a distinct biological parameters regime in which vibration-assisted electron tunnelling occurs. The strong coupling between the spike protein and the receptor enables the vibronic mode to facilitate electron transfer, leading to an increase in the overall transfer probability and consequently augmenting the virus infection dynamics on the microscopic level. This finding emphasizes the importance of understanding the effects of the coupling strength on the interaction between the spike protein and the ACE2 receptor, as it directly influences the electron transfer process and the subsequent infection dynamics.This seemingly straightforward act of electron transfer serves as a crucial molecular switch, facilitating molecular recognition through the detection of vibrational spectra. Specifically, it enables the identification of a virus’s spike protein via its vibronic mode. Research detailed in reference^21^ thoroughly demonstrates that the electron transfer rates can significantly vary in the presence or absence of odorant molecules, depending on certain parameter values. This variation underscores how a basic electron transfer process can trigger the molecular switch, enhancing the lock and key mechanism for molecular interaction. The persistence of quantum coherence in electron transfer processes involving the ACE2 receptor and the spike protein is underpinned by a confluence of biological factors. Conformational dynamics play a crucial role, as the specific three-dimensional structures and the inherent flexibility of these proteins ensure their precise alignment and proximity, thereby facilitating quantum tunneling^34^. This alignment is further modulated by dynamic conformational changes, driven by vibrational modes, which can adjust the tunneling rates and electron transfer efficiency. Electron donor and acceptor sites within the proteins, characterized by specific amino acids or cofactors, are essential for creating favourable conditions for tunneling, with their electronic properties-such as energy levels and spatial orientation-being critical for the quantum mechanical transfer of electrons^35^. The vibronic coupling between electronic states and particular vibrational modes within the spike protein can either enhance or suppress tunneling rates, offering the requisite energy to sustain quantum coherence amidst the biological complexes^36^..

**Figure 4:**
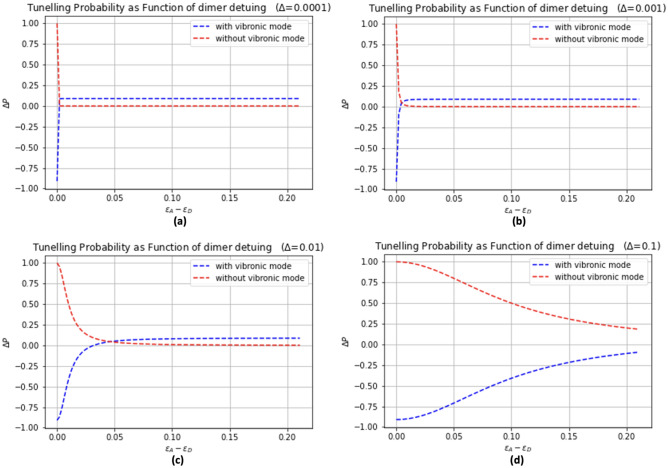
Comparison of electron transfer probabilities with and without the vibronic mode as a function of dimer detuning. (**a**) Transfer probabilities without the vibronic mode, showing a relatively uniform behavior across the range of dimer detuning values. (**b**) Transfer probabilities with the vibronic mode, illustrating the influence of the vibronic mode on the electron transfer probability as the dimer detuning changes. The results emphasize the importance of the vibronic mode in modulating electron tunneling dynamics and reveal its potential role in the infection process.

In Fig. 4a–d, we provide further support for our findings by presenting the observed effects when plotting the maximum transfer probability against the dimer detuning, considering both cases with and without the vibronic mode. These results shed light on the significance of the vibronic mode on the transfer probability within different coupling regimes. In the figures, we depict the maximum transfer probability as a function of the dimer detuning, where the red dashed line represents the case without the vibronic mode, while the blue line represents the inclusion of the vibronic mode. The comparisons between these two cases highlight the varying impact of the vibronic mode on the transfer probability in the presence of different levels of coupling between the donor and acceptor. Specifically, we observe that the influence of the vibronic mode is more pronounced in the intermediate coupling regime. In this regime, the electron transfer process experiences a more significant modulation in the presence of the vibronic mode, resulting in notable deviations in the transfer probability. Conversely, in the weak coupling regime, the impact of the vibronic mode on the transfer probability is relatively subdued, as the electron transfer process is less affected by the vibronic coupling. In the strong coupling regime, however, we observe a contrasting behavior. The influence of the vibronic mode becomes more adverse, leading to a negative transfer probability. This implies that the presence of the vibronic mode hampers the efficiency of the electron transfer process, potentially hindering the desired charge transfer between the donor and acceptor. These findings contribute to our understanding of the intricate interplay between the vibronic mode and the electron transfer dynamics. By examining the maximum transfer probabilities across different coupling regimes, we discern that the impact of the vibronic mode on the transfer probability varies significantly depending on the strength of the donor-acceptor coupling. This insight has implications for designing and optimizing systems that involve electron transfer processes, providing valuable guidance for controlling and enhancing charge transfer efficiencies.

**Figure 5:**
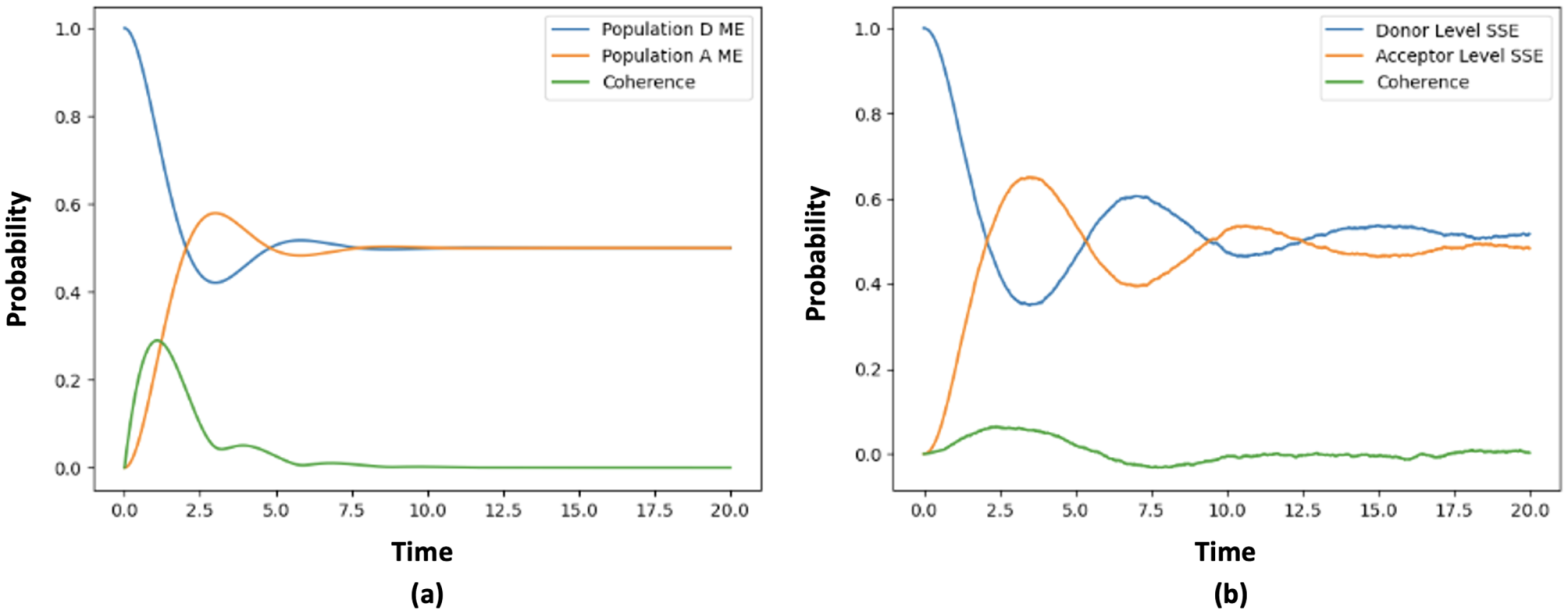
Comparison of simulation results between the (**a**) Master equation and (**b**) the Non-linear Stochastic Schrödinger equation(SSE). The population and coherence dynamics obtained from both methods show a strong agreement within the biological parameters window. This agreement demonstrates the validity of our SSE approach for modeling the microscopic dynamics of the COVID-19 infection process and highlights its potential for further investigation and analysis.

In addition, we conducted a comparative analysis between the numerical simulations of the dynamics based on the Master equation (ME), as presented in the reference,^31^ and our own simulations using the non-linear stochastic Schrödinger equation (SSE). The obtained results regarding the populations and coherence of the dynamics are depicted in Fig. 5. Figure 5a portrays the population and coherence profiles achieved by simulating the Master equation described in reference.^31^ On the other hand, Fig. 5b represents the corresponding outcomes obtained by numerically simulating the non-linear stochastic Schrödinger equation (Eq. 28) and computing the density matrix via ensemble averaging using (Eq. 8) . The comparison between these two sets of simulations showcases a remarkable agreement in terms of population and coherence within the given parameters range. This congruence provides substantial validation for the utilization of our non-linear stochastic Schrödinger equation as a viable modeling approach for comprehending the microscopic dynamics underlying the virus infection as a whole using the Olfaction as biological process and particular in the COVID-19 infection. It is essential to note that further examination of the specific parameters, methodologies, and findings presented in the aforementioned reference^31^ is necessary to fully ascertain the extent and implications of the concurrence observed between the two models.

Furthermore, our numerical results demonstrate the effectiveness of using the quantum state diffusion approach to solving the non-linear Stochastic Schrödinger equation for modelling the dynamics of the COVID-19 infection. The results reveal the importance of vibration-assisted electron tunnelling in the infection process and provide insights into the specific biological parameters regime where this phenomenon occurs. By understanding these dynamics, we hope to contribute to the ongoing research efforts in understanding the biological processes of virus infection by combating the COVID-19 pandemic and inform the development of more effective treatments and preventive measures. The limitations and future research directions discussed in this section also provide a road map for further exploration of the subject matter, ultimately aiming to enhance our knowledge and understanding of the COVID-19 infection dynamics on a microscopic level.

## Conclusions

In this research paper, we have investigated the dynamics of the COVID-19 infection using the quantum state diffusion approach to solving the non-linear Stochastic Schrödinger equation. Our primary objective was to explore how the vibration-assisted electron tunneling in the relevant biological parameters regime augments the virus infection on a microscopic level. By simulating the non-linear Stochastic Schrödinger equation and analyzing the numerical results, we have gained valuable insights into the role of vibration-assisted electron tunneling in the infection process and its dependence on the coupling strength between the virus spike protein and the ACE2 receptor.

Our study has revealed the significance of the coupling strength between the spike protein and the receptor as well as the coupling between the donor and acceptor level of the dimer in facilitating electron transfer and subsequently influencing the infection dynamics. The numerical results demonstrate that for the a weak interaction between the system (spike protein+ ACE2 receptor) and the environment (membrane) and as the dimer( ACE2 receptor) coupling strength increases, the effect of the vibronic mode on the transfer probability becomes more pronounced, leading to a higher transfer probability in the intermediate coupling regime between the dimer and negative effect in the strong coupling. This finding highlights a distinct biological parameters regime in which vibration-assisted electron tunneling occurs and emphasizes the importance of understanding these dynamics in the context of the COVID-19 infection process.

The strong agreement between the simulation results of our stochastic Schrödinger equation and the master equation presented in the references validates the use of our approach in modeling the dynamics of the COVID-19 infection at the microscopic level. Moreover our model shows more significant effect of the dynamics which is more close to the reality. This research contributes to the ongoing efforts to combat the COVID-19 pandemic by deepening our understanding of the infection process and providing insights that could potentially inform the development of more effective treatments and preventive measures.

The interaction between viruses, such as SARS-CoV-2, and host cells through molecular recognition and binding is a critical area of research, potentially paving the way for novel COVID-19 treatments. One approach under consideration involves the use of ACE2 inhibitors and angiotensin receptor blockers (ARBs). ACE2 inhibitors aim to prevent the production of angiotensin proteins, whereas ARBs block the action of these proteins. Interestingly, evidence suggests that ARBs might offer some protection against SARS-CoV-2 by inhibiting the virus’s binding to ACE2 receptors. The introduction of soluble ACE2 is another strategy being explored, which could neutralize the virus by binding to its spike protein before it attaches to membrane bound ACE2 receptors. This complex interaction underscores the potential for targeting the ACE2 pathway and related angiotensin receptors as a therapeutic strategy against COVID-19. Beyond ACE2 and angiotensin receptors, G-protein coupled receptors (GPCRs) represent another significant focus due to their role in various physiological processes and as major pharmaceutical targets. The spike protein of SARS-CoV-2, a glycoprotein, raises questions about its affinity for GPCRs and the potential for novel therapeutic interventions via GPCR agonists. Screening for GPCR agonists has shown promise in identifying compounds with antiviral effects against viruses like Ebola and Marburg. Additionally, the role of neurotransmitters and their precursors, such as tryptophan and serotonin, along with SSRIs and other antidepressants, have been explored for their potential therapeutic effects against SARS-CoV-2. This research opens avenues for using vibrational spectroscopy to identify ligands that could interfere with the virus’s ability to bind to host cells, suggesting a broader application of vibrational theory in developing new treatments for COVID-19.

In conclusion, our research has successfully demonstrated the application of the quantum state diffusion approach to solving the non-linear Stochastic Schrödinger equation in the context of the COVID-19 infection dynamics. The results emphasize the importance of vibration-assisted electron tunneling in the infection process and shed light on the specific biological parameters regime where this phenomenon occurs. By understanding these dynamics, we can further contribute to the global research efforts aimed at combating the COVID-19 pandemic, ultimately leading to the development of improved therapies and preventive strategies to mitigate the impact of this virus on human health and society.

## Data Availability

The data and parameters utilized and analyzed in this research are documented within the published papers. Additionally, the data sets are available from the corresponding author upon a justified request.

## Appendix: Theoratical derivation

### Linear stochastic Schrodinger equation

The dynamics of a quantum open system, characterized by its Hamiltonian, is dictated by the Schrödinger equation.

31 $$\begin{aligned} \frac{d}{d t}| \Psi (t)\rangle = -\frac{i}{\hbar } H_{\text {T}} | \Psi (t) \rangle \end{aligned}$$

The system is coupled to a reservoir comprised of set of bosonic oscillators, as represented by the Hamiltonian $H_R= \sum _{k} \omega _k b_k^+b_k$. The reservoir is best described using the Bargmann coherent state basis, denoted as $|z\rangle = \bigotimes _k |z\rangle$. This basis is a tensor product, bringing together all the distinct oscillators in the environmental configuration. Within this context, the notation $|z\rangle$ is specifically described by the relationship $b_k |z\rangle = z_k |z\rangle$, pointing to the kth mode in the environment. Given this basis, one can view the system’s state vector as a stochastic path or trajectory. Its dynamics in the interaction picture, follows a formula where the standard quantum unit $\hbar$ is taken as unity.

32 $$\begin{aligned} \frac{d}{d t} \langle z | \Psi (t) \rangle = -i \langle z | H_{\text {T}} | \Psi (t) \rangle =(-i H_S + \gamma L^\dagger z^* t) \langle z | \Psi (t) \rangle - i \gamma \sum _k g_k^* e^{-i \omega _k t} \langle z | b_k | \Psi (t) \rangle \end{aligned}$$

Here, the complex Gaussian noise is represented as $z_t^* = -i\sum _k g_k z_k^* e^{i \omega _k t}$, and it conforms to the stipulated auto-correlation function $\alpha (t,s) = \mathscr {M}[z^*(t)z(s)]$. The operator denoted by $\mathscr {M}$ encompasses an ensemble average over the classical noise emanating from $z_t$, and is symbolized as $\mathscr {M} = \int d\mu (z)[\cdot ]$. The integration measure $d\mu (z) = \frac{d^2z}{\pi } e^{-|z|^2}$ encapsulates the classical probabilistic distribution of *z*, while $\int d\mu (z) |z\rangle \langle z| = I$ asserts the completeness condition. Moreover, the time-evolution operator *U*(*t*), governing the interplay between the system and the environment, is defined through $|\Psi (t)\rangle = U(t)|\Psi (0)\rangle$. This operator adheres to an evolution equation that characterizes the dynamical progression.

33 $$\begin{aligned} \frac{d}{d t}U(t) = -i(H_S + \gamma L \sum _k g_k b_k^\dagger e^{i\omega _k t} + \gamma L^\dagger \sum _k g_k^* b_k e^{-i\omega _k t}) U(t) \end{aligned}$$

In situations involving the initial entanglement of the system and environment, the density matrix can be disentangled into a basis of product states. Nonetheless, for the sake of analytical tractability, we suppose that the system and environment are initially separable at outset, i.e., at $t=0$, with the environment occupying its vacuum state: $|\Psi (0)\rangle = |\psi _0\rangle \otimes |0\rangle$. This holds paramount significance to evaluate the term in question.

34 $$\begin{aligned} \langle z | b_k | \Psi (t) \rangle = \langle z | b_k U(t) | 0 \rangle | \psi _0 \rangle \end{aligned}$$

The (Eq. 34) can be computed by two equivalent methods, the Heisenberg and quantum-state diffusion approaches. Firstly we consider the operator in the Heisenberg picture and its equation of motion is takes the form

35 $$\begin{aligned} \frac{d b_k(t)}{d t} = U^\dagger (t)(-i\gamma g_k e^{i\omega _k t}L)U(t) = -i\gamma g_k e^{i\omega _k t}L(t) \end{aligned}$$

The formal solution to the aforementioned equation is as follows:

36 $$\begin{aligned} b_k(t) = b_k - i\gamma g_k \int \limits _0^t ds , e^{i\omega _k s} L(s) \end{aligned}$$

substituting the above solution into the (EQ. 34)

37 $$\begin{aligned} \langle z | U(t) b_k(t) | 0 \rangle = -i\gamma g_k \int \limits _0^t ds, e^{i\omega _k s} \langle z | U(t) L(s) | 0 \rangle \end{aligned}$$

Hence, the (Eq. 32) we have

38 $$\begin{aligned} \frac{d}{d t} |\psi _t(z)\rangle = \left( -i H_S + \gamma L z_t \right) |\psi _t(z)\rangle - \gamma ^2 L^\dagger \int \limits _0^t ds\ \alpha (t,s) \langle z | U(t) L(s) | 0 \rangle |\psi _0\rangle \end{aligned}$$

In the context of a zero-temperature environment, the correlation function is given by $\alpha (t,s) = \sum _{k} |g_k|^2 e^{-i\omega _k(t-s)}$. By utilizing the chain rule on the functional derivative associated with the Gaussian stochastic process, denoted by $z_t$, we can reformulate the equation previously identified as (Eq. 34) in the following manner:

39 $$\begin{aligned} \langle z | b_k U(t) | 0 \rangle = \frac{\partial }{\partial z_k} \langle z | U(t) | 0 \rangle = \int \limits _0^t ds \frac{\partial z_s}{\partial z_k} \frac{\delta }{\delta z_s} \langle z | U(t) | 0 \rangle \end{aligned}$$

and the Stochastic Schrodinger Equation is

40 $$\begin{aligned} \frac{d}{d t} \psi _t(z) = \left( -iH_S + \gamma L z_t - \gamma ^2 L^\dagger \int \limits _0^t ds \alpha (t,s) \hat{X}(t, s, z)\right) \psi _t(z) \end{aligned}$$

by defining an operator $\hat{X}(t,s,z)$

41 $$\begin{aligned} \hat{X}(t, s, z)\psi _t \equiv \dfrac{\delta \psi _t}{\delta z_s} \end{aligned}$$

Here $X(s, s, z) = L$. The consistency condition is used to calculate the time-dependence of *X*(*s*, *s*, *z*).

42 $$\begin{aligned} \frac{d}{dt} \frac{\delta }{\delta z} \psi _t = \frac{\delta }{\delta z} \frac{d}{dt} \psi _t \end{aligned}$$

The equation labeled as (Eq. 40) is governed by linearity. In the case of an infinite heat bath, the norm $||\psi _t||$ of the solution converges to zero with a probability of one, while it diverges to infinity with a probability of zero. This non-Markovian development aligns with the principles of the reduced density operator technique. By aggregating the outcomes from (Eq. 34) relative to the noise $z_t$ , we can accurately reconstruct the corresponding density matrix of the system.

43 $$\begin{aligned} \rho _t \equiv \textrm{Tr}_ {\textrm{env}} \left[ e^{-iH{\textrm{T}}t} |\psi _0\rangle \langle \psi _0| \otimes \rho _{\textrm{env},0} e^{iH_{\textrm{T}}t} \right] = \mathscr {M} \left[ |\psi _t(z)\rangle \langle \psi _t(z)|\right] . \end{aligned}$$

### Non-linear non-markovain quantum state diffusion

The non-Markovain Unraveling of Quantum State Diffusion (QSD) predicated on states with normalization

44 $$\begin{aligned} \tilde{\psi }_t(z) = \frac{\psi _t(z)}{||\psi _t(z)||} \end{aligned}$$

could be achieved using the Girsanov transformation^24^.

45 $$\begin{aligned} \frac{d}{dt}\tilde{\psi }_t = -iH_S\tilde{\psi }_t + (L - \langle L\rangle _t)\tilde{\psi }_t \tilde{z}_{t} - \int \limits _0^t ds \alpha (t,s) \langle (L^\dagger - \langle L^\dagger \rangle _{s}) \hat{X}(t,s,{\tilde{z}_t}) - (L^\dagger - \langle L^\dagger \rangle _{s})\hat{X}(t,s,{\tilde{z}_t}) \rangle \tilde{\psi }_t \end{aligned}$$

Where ${\tilde{z}_t}$ is the shifted noise,

46 $$\begin{aligned} {\tilde{z}_t}=z_t+\int \limits _0^t ds \alpha (t,s) \langle L^\dagger \rangle _{s} \end{aligned}$$

and $\langle L\rangle _{s}=\langle \tilde{\psi }_t| L |\tilde{\psi }_t \rangle$ is the quantum average.

The non-linear non-markovain QSD equation can have the compact form by introducing $\Delta _t(A)=A-\langle A\rangle _t$

47 $$\begin{aligned} \frac{d}{dt}\tilde{\psi }_t = -iH_S\tilde{\psi }_t + \Delta _t(L)\tilde{\psi }_{t}z^{*}_t - \Delta _t(L^{\dagger })\bar{X}(t, \tilde{z})\tilde{\psi }_t + h\Delta _t(L^{\dagger })\bar{X}(t, \tilde{z})_t\tilde{\psi }_t \end{aligned}$$

Where

48 $$\begin{aligned} \bar{X}(t, z) = \int \limits _{0}^{t} \alpha (t,s)\hat{X}(t,s,z)ds \end{aligned}$$

The (Eq. 45or  47) is fundamental non-Markovain Quantum state diffusion and in the next section perturbative treatment start with this equation. Numerical simulations of the some of the non-Markovain open quantum system dynamics can be found in this paper.^25^ In this paper we focus on the Markovain dynamics which can be obtained from the (Eq. 45 or  47) by simply replacing $\alpha (t,s)\rightarrow \delta (t-s)$^26^. To obtain the non-Markovian equations, specific assumptions were made. First, the environment exhibits bosonic characteristics, and the initial state of both the system and the environment is distinguishable: $\rho _0 = \rho _S^{(0)} \otimes \rho _{env}^{(0)}$, which remains unaffected by the noise $z_t$. Second, the non-Markovian equations are exclusively derived from the underlying microscopic model, as evident in the Hamiltonian presented in (Eq. 1).

### Time-dependent perturbation theory

This section delves into the non-Markovian Quantum State Diffusion (QSD) approach as a valuable technique for addressing quantum systems affected by non-Markovian effects. However, the determination of the operator $\hat{X}$ in (Eq. 45) can pose challenges, and the introduction of nonlocal noise in the foundational (Eq. 45 or Eq. 47) can render numerical simulations intricate. To overcome these challenges, this section introduces a formal perturbation scheme for the non-Markovian QSD equation.

To computationally solve the non-Markovian QSD equation (Eq. 48), it necessitates knowledge of the operator $\hat{X}(t, s, z)$ derived from (Eq. 41). Nevertheless, since $\hat{X}$ exclusively emerges in (Eq. 45) under the memory integral $\int \limits _{0}^{t} \alpha (t,s)\hat{X}(t,s,z)ds$, and if the environmental correlation time remains relatively short, solely s-values proximate to the upper limit of integration hold significance. Consequently, we can expand the operator $\hat{X}(t, s, z)$ in (Eq. 41) using a series expansion in powers of $(t-s)$.

49 $$\begin{aligned} \hat{X}(t,s,z) = \hat{X}(s,s,z) + \frac{\partial \hat{X}(t,s,z)}{\partial t} \Bigg |_{t=s} (t-s) + \frac{1}{2} \frac{\partial ^2 \hat{X}(t,s,z)}{\partial t^2} \Bigg |_{t=s} (t-s)^2 + \dots \end{aligned}$$

Incorporating the operator $\hat{X}(t, s, z)$, as expounded in (Eq. 48), within the framework of the non-Markovian Quantum State Diffusion (QSD) equation, as referenced in either (Eq. 45 or Eq. 47), yields a set of refined QSD equations. The reliability and applicability of these resultant equations are intimately connected to the environmental correlation time, denoted as $\tau$. The formal expansion of the non-Markovian QSD equation, presented (Eq. 49), is structured around the dimensionless parameter $\omega \tau$. Here, $\omega$ signifies a characteristic system frequency, while $\tau$ characterizes the correlation time of the environment. Remarkably, as the correlation time $\tau$ approaches zero, the primary equation seamlessly aligns with the conventional Markov Quantum State Diffusion, marking a transition from non-Markovian to Markovian dynamics. However, as one progresses to the next order in the equation, significant modifications to Markovian behaviors become evident. Subsequently, this discourse will delve into the intricacies of this approximate formulation, with particular emphasis on its first-order implications.

In the pursuit of determining the value of the operator $\hat{X}(t, s, z)$ at $t = s$, it is noteworthy that an exhaustive definition is not necessarily required. Leveraging the consistency condition elucidated in Equation (Eq. 42), specific formulations for $\hat{X}(t, s, z)$ can be derived, providing a succinct and tractable approach to its evaluation.

50 $$\begin{aligned} \hat{X}(s,s,z)= & {} L \end{aligned}$$

51 $$\begin{aligned} \frac{\partial \hat{X}(t,s,z)}{\partial t}\Big |_{t=s}= & {} -i[H, L] - \int \limits _0^s \alpha (s,u)du [L^\dagger , L]L \end{aligned}$$

The non-Markovian QSD equation up to the first order can be written by substituting the first two terms of the expansion (Eq. 49) into (Eq. 48). Here, *H* represents the Hamiltonian, while *L* denotes the Lindblad operator. The resulting expression for $\bar{X}$ involves (Eq. 50) and (Eq. 51).

52 $$\begin{aligned} \hat{X}(t)= & {} g_0(t)L - g_1(t)i[H, L] - g_2(t)[L^{\dagger }, L]L \end{aligned}$$

53 $$\begin{aligned} g_0(t)= & {} \int \limits _0^t \alpha (t,s) ds \end{aligned}$$

54 $$\begin{aligned} g_1(t)= & {} \int \limits _{0}^{t} \alpha (t,s)(t-s)ds \end{aligned}$$

55 $$\begin{aligned} g_2(t)= & {} \int \limits _0^t \int \limits _0^s \alpha (t,s) \alpha (s,u) (t-s) du ds \end{aligned}$$

56 $$\begin{aligned} \frac{d}{dt}\tilde{\psi }_t= & {} -iH_S\tilde{\psi }_t + \Delta _t(L)\tilde{z}_t - g_0(t)((\Delta _t(L^{\dagger })L - \langle \Delta _t(L^{\dagger })L \rangle _t))\tilde{\psi }_t + ig_1(t)(\Delta _t(L^{\dagger })[H,L] - \langle \Delta _t(L^{\dagger })[H,L]\rangle _t))\tilde{\psi }_t\nonumber \\{} & {} + g_2(t)((\Delta _t(L^{\dagger })[L^{\dagger },L]L - \langle \Delta _t(L^{\dagger })[L^{\dagger },L]\rangle _t))\tilde{\psi }_t \end{aligned}$$

In the realm of non-Markovian Quantum State Diffusion (QSD) equations, the zeroth-order term, obtained through expansion, introduces a crucial magnitude that is contingent upon the typical relaxation rate of the quantum system, symbolized as $\Gamma$. When one delves into the first-order terms, they emerge as corrections characterized by scaling factors of either $\omega \tau$ or $\Gamma \tau$, where $\omega$ signifies the standard system frequency represented by the Hamiltonian *H*, and the composite operator $L^{\dagger }L$ embodies a common system relaxation rate, $\Gamma$. In situations marked by non-Markovian dynamics, wherein the environmental correlation time remains finite but does not exceed the characteristic time scales of the quantum system, it is anticipated that the expanded QSD equation maintains its validity. However, in the limit where the correlation time $\tau$ tends towards zero, the Markovian regime emerges. In this limiting scenario, only the zeroth-order term persists, culminating in the well-established Markov Quantum State Diffusion equation that governs the quantum dynamics for $t > 0$. This delineation underscores the transition from non-Markovian to Markovian behavior in quantum systems and offers valuable insights into the conditions under which each regime prevails.

The non-Markovian attributes of a quantum system manifest through time-dependent coefficients that undergo rapid changes on a timescale aligned with the environmental correlation time. The initial-order expansion does not encompass noise, while higher-order expansions introduce noise components. Notably, the approximate non-Markovian Quantum State Diffusion (QSD) equation (Eq. 56) conserves the norm of the wave function. The primary challenge associated with using non-Markovian unravelings stems from the presence of non-local noise within the functional derivative or the integrand operator. Nevertheless, the approximate QSD equation simplifies the non-Markovian QSD equation (Eq. 56). The effectiveness of the non-Markovian QSD approach depends on the determination of the operator $\hat{X}(t, s, z)$.
